# Left ventricular longitudinal strain in patients with type 2 diabetes mellitus is independently associated with glycated hemoglobin level

**DOI:** 10.1002/clc.24136

**Published:** 2023-08-24

**Authors:** Jinmei Gao, Min Xu, Mingxia Gong, Shu Jiang, Zhenni Yang, Xiaohong Jiang, Ming Chen

**Affiliations:** ^1^ Department of Echocardiography and Cardiology The Third Affiliated Hospital of Soochow University Chang Zhou China; ^2^ Department of Endocrinology The Third Affiliated Hospital of Soochow University Chang Zhou China; ^3^ Department of Medical Imaging The Third Affiliated Hospital of Soochow University Chang Zhou China

**Keywords:** diabetes mellitus type 2, glycated hemoglobin, subclinical left ventricular myocardial injury, systolic function, two‐dimensional speckle tracking imaging

## Abstract

**Objective:**

Left ventricular and left atrial strain are sensitive and reliable markers for evaluating cardiac function in patients with type 2 diabetes mellitus (T2DM), with interactions between the two parameters. The present study aimed to analyze the correlation between global longitudinal strain (GLS) of the left ventricle and glycated hemoglobin (HbA1c) levels in patients with T2DM.

**Methods:**

A total of 292 patients clinically diagnosed with T2DM were selected and divided into three groups according to HbA1c level. The strains of the left atrium and left ventricle in the three groups of T2DM patients with different HbA1c levels were compared. Univariate and multivariate (including left atrial functional indicators) linear regression analyses were performed to assess the relationship between strain indicators and HbA1c levels. Generalized additive models were used to examine the relationship between strain indicators and HbA1c levels.

**Results:**

There were significant differences among the three groups in terms of age, microalbuminuria, total cholesterol, fasting blood glucose, postprandial blood glucose, and HbA1c level, and left atrial conduit longitudinal strain (LAScd) and GLS (*p* < .05). Univariate and multivariate linear regression analyses revealed that, as HbA1c levels increased, the absolute value of GLS gradually decreased (*p* < .001). Curve fitting revealed a positive correlation between HbA1c level and GLS, which was not affected by left atrial function.

**Conclusion:**

Left ventricular GLS was independently correlated with HbA1c level in patients with T2DM and was not affected by left atrial function.

## Graphical abstract

Among T2DM patients with preserved LVEF, left ventricular GLS, an essential indicator reflecting subclinical changes in the left ventricle, exhibited a positive correlation with increasing HbA1c levels and was not influenced by left atrial function.

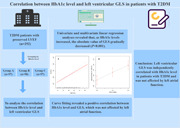

## 1 INTRODUCTION

Diabetes is a metabolic disease caused by genetic and environmental factors, leading to insulin insensitivity, insulin deficiency, and impaired biological function. Due to its high prevalence and associated disability and mortality rates, it has become a serious global health problem.^1^ Studies have shown that type 2 diabetes mellitus (T2DM) is associated with hyperglycemia, insulin resistance, and insulin deficiency, all of which contribute to accelerated atherosclerosis and increased incidence of cardiovascular diseases.^2^ Diabetes can also cause changes in cardiomyocytes at the molecular and cellular levels, leading to left ventricular (LV) dysfunction, even in the absence of coronary artery disease and hypertension, and may present without symptoms.^3^, ^4^ Research has demonstrated that the risk for heart failure (HF) among patients with diabetes is more than twice that of those without the disease.^5^ Moreover, HF with preserved ejection fraction (HFpEF) is the most common type of HF among patients with T2DM,^6^ often accompanied by preclinical LV systolic dysfunction.^7^ Diabetic cardiomyopathy (DCM) occurs insidiously, with persistent hyperglycemia slowly affecting the myocardium, manifesting early as HFpEF, which is also known as the subclinical stage of DCM.^8^ Conventional echocardiography and electrocardiographic examinations often fail to yield positive findings, leading to neglect of cardiac protection in clinical diagnosis and treatment.

Conventional echocardiography is a commonly used modality for screening and assessing cardiac structural and functional damage in patients with diabetes; however, LV ejection fraction (LVEF) cannot sensitively reflect changes in LV function. In recent years, the development of noninvasive ultrasound imaging techniques has facilitated the determination of subclinical cardiac function. Global longitudinal strain (GLS) of the left ventricle, determined by speckle tracking, is a useful metric for the early identification of subclinical myocardial contractile dysfunction.^9^ Glycated hemoglobin A1c (HbA1c) plays a central role in the diagnosis, prevention, and monitoring of diabetes,^10^ and studies have shown that higher HbA1c levels can increase the risk for the development of HF. Li et al.^6^ used cardiac magnetic resonance (CMR) imaging to determine LV GLS in patients with T2DM, and showed that HbA1c levels can independently predict LV myocardial deformation in this patient population. Using multivariate regression analysis, Zhang et al. reported that HbA1c levels were independently correlated with LV GLS.^7^ Cardiac changes in patients with diabetes often manifest as increased left atrial volume and decreased left atrial function. Left atrial reservoir longitudinal strain (LASr) is a reliable marker of LV diastolic function^11^; however, previous studies did not consider the impact of left atrial function on the correlation between LV GLS and HbA1c levels. As such, the present study aimed to investigate the correlation between LV GLS and HbA1c in patients with T2DM after considering the impact of left atrial function, to find a sensitive indicator for early identification of cardiac dysfunction in diabetic patients, which could play an active role in optimizing and adjusting treatment strategies and preventing HF.

## 2 MATERIALS AND METHODS

### 2.1 Study subjects

Data were selected from a consecutive cohort of 302 patients diagnosed with T2DM, who were hospitalized in the Department of Endocrinology at The First People's Hospital of Changzhou (Jiangsu, China) from January to December 2021. Inclusion criteria were as follows: Diagnosis of T2DM according to the American Diabetes Association standards^12^: having a fasting plasma glucose (FPG) level of ≥7.0 mmol/L; a 2‐h plasma glucose (PG) level of ≥11.1 mmol/L during an oral glucose tolerance test (OGTT); an HbA1c level of ≥6.5%; age ≥18 years; and complete clinical data. Exclusion criteria were as follows; type 1 diabetes or gestational diabetes; diagnosed with coronary artery disease or other cardiac disease history based on coronary computed tomography (CT) or angiography; valvular heart disease; LVEF < 50%; atrial fibrillation or severe arrhythmia affecting image recording; poor image quality on transthoracic echocardiography; and incomplete clinical data.

## 3 METHODS

### 3.1 Data collection

General patient data, including age, sex, body mass index (BMI), duration of diabetes, systolic blood pressure, diastolic blood pressure, heart rate, and history of hypertension and coronary artery disease, were collected. Biochemical analysis was performed on all subjects using standard laboratory techniques. We collected blood samples from patients on the second morning of admission on an empty stomach. We used a fully automatic biochemical analyzer (Beckman automatic biochemical analyzer, DM‐AU5800) to measure FPG, HbA1c, total cholesterol (TC), triglycerides (TG), high‐density lipoprotein (HDL), low‐density lipoprotein (LDL), microalbuminuria, creatinine, urine albumin‐to‐creatinine ratio (UACR), and brain natriuretic peptide (BNP).

### 3.2 Routine echocardiography

A color Doppler ultrasound diagnostic device equipped with M5S and 4V probes (General Electric Medical Systems, Norway) were used. The echocardiogram examination was simultaneously completed on the second day of hospitalization. All subjects were positioned in the left lateral decubitus position and encouraged to breathe calmly while electrocardiogram was recorded simultaneously. Simpson's biplane method was used to assess end‐diastolic volume, end‐systolic volume, and LVEF. Standard apical four‐, two‐, and three‐chamber images of the left ventricle, the standard horizontal short‐axis section of the mitral valve, the horizontal short‐axis section of the papillary muscle, and the apical horizontal short‐axis section images were acquired in two‐dimensional (2D) mode. The images of all patients were digitally stored for subsequent analysis. Image acquisition and analysis were completed independently by two attending physicians, and the averaged values were taken. Pulsed‐wave Doppler was used to measure peak velocities of early diastolic (E wave) and late diastolic (A wave) mitral valve flow, and the E/A ratio was calculated. Tissue Doppler imaging was used to measure the early diastolic mitral annulus velocity (e′) and to calculate the E/e′ ratio.

### 3.3 2D speckle‐tracking echocardiography strain analysis

*Calculation of LV GLS*: The AFI component of the EchoPAC 203 version (General Electric Medical Systems, Norway) software package automatically tracked the endocardial and epicardial boundaries of the left ventricle, displaying the 17‐segment 2D strain‐time curve and bull's‐eye plot of the left ventricle. If tracing was poor, the endocardial line was adjusted manually, and the image was reread. If accurate tracking could not be achieved for >2 segments after adjustment, the patient was excluded. The software automatically calculated LV GLS, which was used as a metric to evaluate LV systolic function.

*Measurement of left atrial volume and strain function*: One point was taken at the midpoint of the mitral annulus and another point on the endocardium at the left atrial roof. The system automatically sketched and displayed the left atrial endocardial contour. Local manual adjustments were made so that the depiction fitted the boundary. The software automatically generated a left atrial time‐volume curve, and the analysis calculated left atrial maximum volume (LAVmax), left atrial minimum volume (LAVmin), and left atrial presystolic volume (LAVpre). Meanwhile, LASr, left atrial conduit longitudinal strain (LAScd), and left atrial contraction longitudinal strain (LASct) were calculated.

### 3.4 Statistical analysis

SPSS version 25 (IBM Corporation) and R version 3.4.3 (http://www.R-project.org) were used for data analysis. The data were stratified according to statistics and HbA1c tertiles. Continuous variables are expressed as mean ± standard deviation (SD) or median (interquartile range [i.e., Q_1_–Q_3_]), while categorical variables are expressed as frequency (percentage). The *χ* ^2^ test (for categorical variables), one‐way analysis of variance (for normally distributed continuous variables), and Kruskal–Wallis test (for skewed continuous variables) were used to analyze differences between groups.

To investigate the association between LV GLS and HbA1c level, three different models were constructed using univariate and multivariate linear regression methods, including unadjusted, partially adjusted, and fully adjusted models. In multivariate regression analysis, if the regression coefficient of HbA1c changed by >10% after introducing a factor in the basic model or excluding a factor in the full model, or if the factor was significantly associated with GLS (*p* < .10), it was included as a potential confounder in the final model. The generalized additive model (GAM) was used for curve fitting. Differences with *p* < .05 were considered to be statistically significant.

## 4 RESULTS

### 4.1 Comparisons of general data among the three patient groups

**Figure 1:**
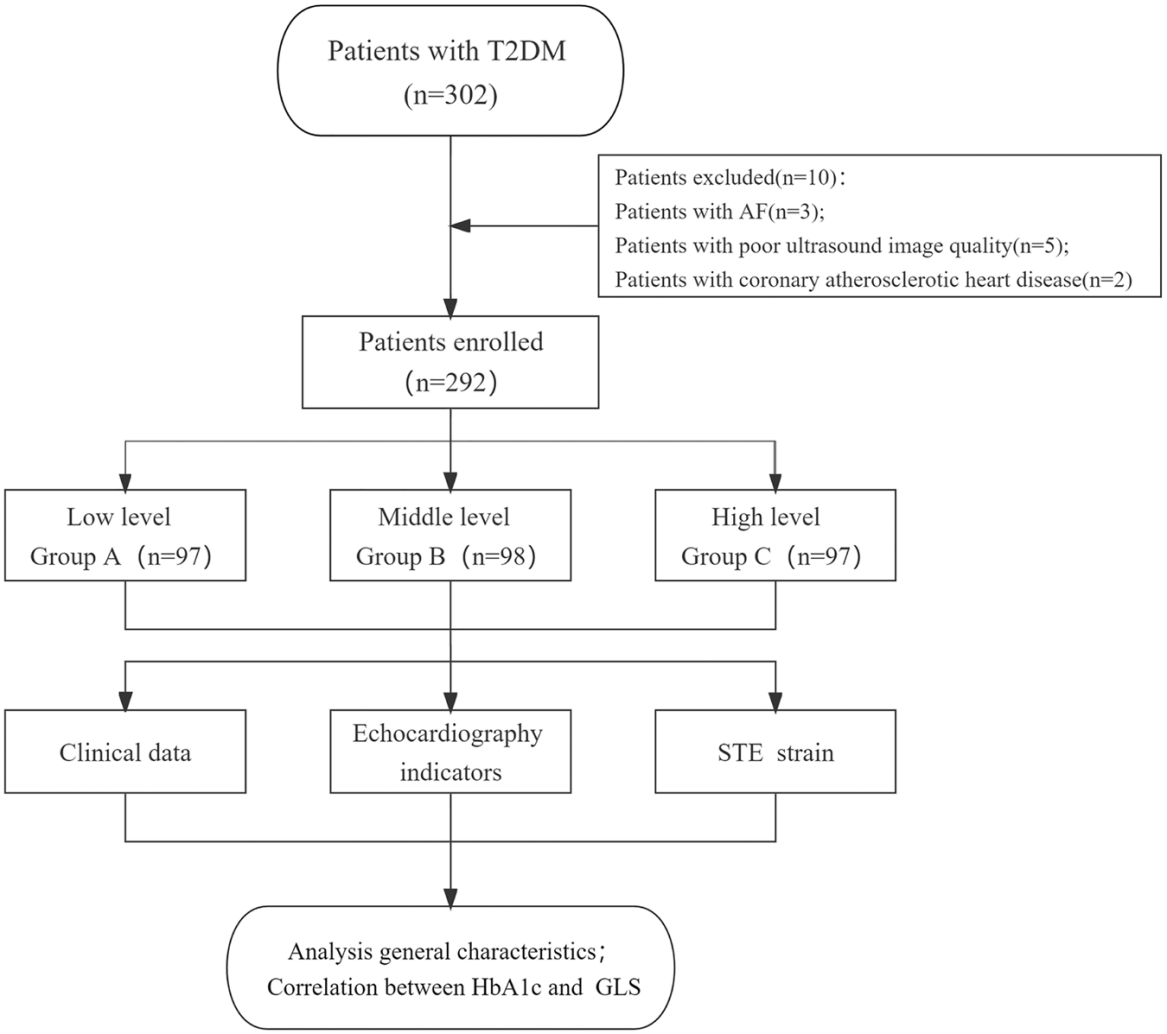
Study scheme. AF, atrial fibrillation; STE, speckle tracking echocardiography; T2DM, type 2 diabetes mellitus.

A total of 302 patients with T2DM were included in this study, 3 patients had atrial fibrillation, 5 patients had poor ultrasound image quality, 2 patients were diagnosed as coronary heart disease, and 292 patients were finally included (Figure 1). A total of 292 patients with T2DM were enrolled in this study and were divided into three equal groups according to their HbA1c levels: Group A (7.3 ± 0.8), 97 patients, the mean age was 61.59 ± 9.10 years, *n* = 48 for men; Group B (9.6 ± 0.5), 98 patients, the mean age was 59.81 ± 10.06 years, *n* = 47 for men; Group C (12.3 ± 1.4), 97 patients, the mean age was 57.91 ± 10.26 years, *n* = 56 for men. The basic clinical characteristics of the three groups are shown in Table 1. Statistically significant differences among the three groups were found in age, microalbuminuria, TC, fasting blood glucose (FBG), postprandial blood glucose (PBG), LAScd, and GLS (all *p* < .05). As HbA1c level gradually increased, the GLS value also gradually increased (−16.4 ± 2.1% vs. −15.5 ± 2.7% vs. −14.2 ± 2.4%; *p* < .001). There was no statistical significance in conventional echocardiographic (LVEF, E/e′ ratio), left atrial volume (LAVmin, LAVmax, LAVpre), and left atrial strain (LASR, LASct) indicators among the three groups (all *p* > .05).

**Table 1:** Comparison of general data, biochemical indicators, and echocardiographic measurements among the three groups (*n* = 292).

Observed indicators	Group A (*n* = 97)	Group B (*n* = 98)	Group C (*n* = 97)	*p* Value
HbA1c (%)	7.3 ± 0.8	9.6 ± 0.5	12.3 ± 1.4	<.001
Men, *n* (%)	48 (52.2%)	47 (47.9%)	56 (54.9%)	.614
Age (years)	61.59 ± 9.10	59.81 ± 10.06	57.91 ± 10.26	.035
Duration of diabetes (years)	8.4 ± 6.9	8.1 ± 6.8	8.0 ± 5.6	.912
Body mass index (kg/m^2^)	24.3 ± 3.1	24.2 ± 3.1	24.8 ± 3.2	.301
Smoking, *n* (%)	11 (11.9%)	14 (14.2%)	20 (19.6%)	.314
Hypertension, *n* (%)	61 (66.3%)	60 (61.2%)	57 (55.9%)	.331
SBP (mmHg)	132.5 ± 11.1	131.2 ± 19.1	131.8 ± 17.1	.870
DBP (mmHg)	79.9 ± 7.1	78.9 ± 12.7	78.5 ± 11.4	.646
DM complications				
DN, *n* (%)	36 (39.1%)	35 (35.7%)	49 (48.0%)	.187
DR, *n* (%)	11 (11.9%)	11 (11.2%)	11 (10.7%)	.967
DK, *n* (%)	14 (15.3%)	18 (18.3%)	16 (15.6%)	.827
Blood examination				
FBG (mmol/L)	8.0 ± 2.3	9.5 ± 2.4	9.5 ± 2.7	<.001
PBG (mmol/L)	12.2 ± 3.2	13.7 ± 3.4	13.2 ± 4.4	.020
Total cholesterol (mmol/L)	4.52 ± 1.12	4.50 ± 1.22	4.91 ± 1.16	.022
Triglycerides (mmol/L)	2.39 ± 2.82	2.56 ± 2.11	3.132 ± 2.82	.116
HDL (mmol/L)	1.10 ± 0.41	1.16 ± 0.44	1.18 ± 0.64	.526
LDL (mmol/L)	2.51 ± 0.89	2.31 ± 1.18	2.51 ± 1.14	.356
Creatinine, mg/dL	84.4 ± 37.5	86.6 ± 39.9	83.3 ± 34.1	.815
microalbumin (mg/L)	7.5 (4.9–14.7)	12.4 (5.8–29.6)	13.1 (5.3–27.2)	.043
UACR (mg/g)	7.0 (2.6–12.7)	13.4 (7.2–28.4)	12.3 (3.9–32.1)	.126
eGFR (mL/min/1.73 m^2^)	78.0 ± 25.8	77.2 ± 28.6	84.8 ± 27.4	.100
BNP, pg/dL	39.0 (21.0–61.5)	40.5 (20.3–66.5)	30.0 (18.0–57.0)	.722
Echocardiographic parameters				
LVEF (%)	58.6 ± 5.4	58.8 ± 5.4	59.0 ± 5.5	.851
LVEDVI (mL/m^2^)	47.7 ± 12.6	47.0 ± 11.8	47.1 ± 11.8	.915
LVESVI (mL/m^2^)	20.2 ± 5.7	19.7 ± 5.8	20.1 ± 5.8	.843
LAVImin (mL/m^2^)	13.4 ± 3.4	12.9 ± 3.7	13.4 ± 3.9	.621
LAVImax (mL/m^2^)	27.7 ± 6.9	27.1 ± 7.7	27.6 ± 7.6	.845
LAVIpre (mL/m^2^)	21.1 ± 6.3	20.2 ± 6.0	20.6 ± 5.8	.544
E (cm/s)	69.5 ± 14.8	69.6 ± 16.0	69.5 ± 15.8	.999
A (cm/s)	91.2 ± 15.5	89.3 ± 14.0	90.3 ± 16.8	.692
e′ (cm/s)	6.7 ± 1.7	6.6 ± 1.8	6.3 ± 1.6	.324
E/e'ratio	9.7 ± 2.5	10.1 ± 2.3	10.1 ± 2.3	.412
LVGLS (%)	−16.4 ± 2.1	−15.5 ± 2.7	−14.2 ± 2.4	<.001
LASr (%)	19.4 ± 3.6	20.0 ± 4.9	20.6 ± 4.8	.157
LAScd (%)	−8.6 ± 3.9	−8.7 ± 3.9	−9.9 ± 4.4	.045
LASct (%)	−11.1 ± 3.4	−11.1 ± 4.3	−10.4 ± 4.0	.414

Abbreviations: A, transmitral Doppler peak late velocity; BNP, B‐type natriuretic peptide; DBP, diastolic blood pressure; DKD, Diabetic kidney disease; DN, diabetic neuropathy; DR, diabetic retinopathy; E, transmitral Doppler early peak velocity; E/e′, ratio of transmitral Doppler early peak velocity (E) and tissue Doppler average early diastolic mitral annulus velocity (e′); eGFR, estimated glomerular filtration rate; FBG, fasting blood glucose; HbA1c, glycated hemoglobin A1c; HDL, high‐density lipoprotein; LAScd, Longitudinal strain of left atrial passage; LASct, Longi‐tudinal strain of left atrium during systole; LASr, Longitudinal strain of left atrial reserve; LAVImax, maximum LA volume index; LAVImin, minimum left atrial volume index; LAVIpre, presystolic LA volume index; LDL, low‐density lipoprotein; LVEF, left ventricular ejection fraction; LVEDVI, left ventricular end‐diastolic volume index; LVESVI, left ventricular end‐systolic volume index; LVGLS, left ventricular global longitudinal strain; PBG, postprandial blood glucose; SBP, systolic blood pressure; UACR, urine albumin‐to‐creatinine ratio.

### 4.2 HbA1c level and subclinical changes in the left ventricle

Univariate linear regression analysis was performed using LV GLS as the dependent variable and HbA1c along with other factors as independent variables. Results indicated that patient BMI, microalbuminuria, LVEF, and FBG were potential factors affecting LV longitudinal strain (GLS) (*p* < .05). Specific data are reported in Table 2.

**Table 2:** Factors associated with global longitudinal strain (GLS) (*n* = 292).

Parameter	Statistics	*β*值	95% CI	*p* Value
Age (years)	59.7 ± 9.9	−.006	(−0.041, 0.029)	.743
Male, *n* (%)	151 (51.7%)	.655	(0.037, 1.342)	.064
Smoking, *n* (%)	45 (15.4%)	.142	(−0.821, 1.106)	.772
Duration of diabetes (years)	8.2 ± 6.5	.017	−0.037, 0.071	.536
Body mass index (kg/m^2^)	24.5 ± 3.2	.120	(0.011, 0.229)	.032
Hypertension, *n* (%)	178 (60.9%)	−.081	(−0.794, 0.632)	.824
SBP (mmHg)	131.9 ± 16.2	−.000	(−0.022, 0.021)	.965
DBP (mmHg)	79.1 ± 10.7	−.007	(−0.039, 0.025)	.669
DM complications				
DR, *n* (%)	33 (11.3%)	.612	(−0.484, 1.709)	.274
DN, *n* (%)	120 (41.1%)	.021	(−0.686, 0.728)	.952
CKD, *n* (%)	48 (16.5%)	.085	(−0.855, 1.024)	.860
Blood examination				
FBG (mmol/L）	9.1 ± 2.6	.171	(0.039, 0.303)	.011
PBG (mmol/L）	13.1 ± 3.8	.048	(−0.044, 0.139)	.307
Total cholesterol (mmol/L)	4.65 ± 1.18	−.042	(−0.337, 0.253)	.780
Triglycerides (mmol/L)	2.71 ± 2.61	.082	(−0.051, 0.215)	.229
HDL (mmol/L)	1.15 ± 0.51	.384	(−0.297, 1.066)	.269
LDL (mmol/L)	2.44 ± 1.08	−.186	(−0.508, 0.136)	.257
Creatinine, mg/dL	84.7 ± 37.1	−.004	(−0.014, 0.005)	.347
microalbumin (mg/L)	30.4 ± 55.0	.007	(0.000, 0.013)	.041
UACR (mg/g)	43.9 ± 106.5	.003	(−0.001, 0.006)	.108
eGFR, mL/min/1.73 m^2^	80.1 ± 27.5	.010	(−0.002, 0.023)	.108
BNP, pg/dL	58.2 ± 73.1	.004	(−0.001, 0.008)	.130
Echocardiographic parameters				
LVEF (%)	58.8 ± 5.4	−.168	(−0.229, −0.106)	<.001
E (cm/s)	69.6 ± 15.56	−.002	(−0.025, 0.020)	.855
A (cm/s)	90.26 ± 15.5	.001	(−0.021, 0.024)	.899
e′ (cm/s)	6.5 ± 1.7	−.107	(−0.308, 0.094)	.296
E/e'ratio	9.9 ± 2.4	.040	(−0.108, 0.188)	.599
LVEDVI (mL/m^2^)	47.3 ± 12.0	−.013	(−0.042, 0.016)	.374
LVESVI (mL/m^2^)	19.9 ± 5.7	.049	(−0.011, 0.110)	.109
LAVImin (mL/m^2^)	13.3 ± 3.7	.019	(−0.075, 0.113)	.696
LAVImax (mL/m^2^)	27.5 ± 7.4	.013	(−0.034, 0.060)	.581
LAVIpre (mL/m^2^)	20.6 ± 6.0	.005	(−0.052, 0.063)	.853
LASR (%)	20.0 ± 4.5	−.011	(−0.089, 0.066)	.772
LASCD (%)	−9.1 ± 4.1	−.019	(−0.104, 0.066)	.655
LASCT (%)	−10.9 ± 3.4	.037	(−0.051, 0.126)	.408

Abbreviations: A, transmitral Doppler peak late velocity; BNP, B‐type natriuretic peptide; DBP, diastolic blood pressure; DKD, Diabetic kidney disease; DN, diabetic neuropathy; DR, diabetic retinopathy; E, transmitral Doppler early peak velocity; E/e′, ratio of transmitral Doppler early peak velocity (E) and tissue Doppler average early diastolic mitral annulus velocity (e′); eGFR, estimated glomerular filtration rate; FBG, fasting blood glucose; HbA1c, glycated hemoglobin A1c; HDL, high‐density lipoprotein; LAScd, Longitudinal strain of left atrial passage; LASct, Longi‐tudinal strain of left atrium during systole; LASr, Longitudinal strain of left atrial reserve; LAVImax, maximum LA volume index; LAVImin, minimum left atrial volume index; LAVIpre, presystolic LA volume index; LDL, low‐density lipoprotein; LVEF, left ventricular ejection fraction; LVEDVI, left ventricular end‐diastolic volume index; LVESVI, left ventricular end‐systolic volume index; LVGLS, left ventricular global longitudinal strain; PBG, postprandial blood glucose; SBP, systolic blood pressure; UACR, urine albumin‐to‐creatinine ratio.

### 4.3 Influence of HbA1c level on subclinical LV systolic function

The results of univariate and multivariate linear regression analyses examining the relationship between LV GLS and HbA1c (continuous variable and tertile groups) are summarized in Table 3. The unadjusted model corresponded to univariate linear regression analysis; the preliminary adjustment model incorporated age, sex, BMI, duration of diabetes, TC, FBG, PBG, microalbuminuria, and LVEF; and the fully adjusted model included age, sex, BMI, duration of diabetes, TC, FBG, PBG, microalbuminuria, LVEF, and LAScd. For continuous HbA1c levels, according to the regression equations of unadjusted, preliminary adjusted, and fully adjusted regression, as HbA1c levels increased, GLS values also gradually increased (*β* = .372; *β* = .370; *β* = .376; all *p* < .001). In the HbA1c tertile groups, as HbA1c levels increased, GLS values also gradually increased (all *p* < .001).

**Table 3:** Linear regression analysis of the relationship between LV GLS and glycated hemoglobin level.

	The unadjusted model		The preliminary adjustment model		The fully adjusted model	
	*β* (95%)	*p* Value	*β* (95%)	*p* Value	*β* (95%)	*p* Value
HbA1c (%)	0.372 (0.244,0.501)	<.0001	0.370 (0.232,0.509)	<.0001	0.376 (0.252,0.500)	<.0001
Group A	0		0		0	
Group B	0.864 (0.158,1.570)	.017	0.896 (0.162, 1.629)	.0175	0.846 (0.148, 1.544)	.0184
Group C	2.211 (1.511,2.910)	<.0001	2.273 (1.531, 3.016)	<.0001	2.130 (1.453, 2.807)	<.0001

*Note*: The unadjusted model corresponds to the univariate linear regression analysis; the preliminary adjustment model included age, sex, body mass index (BMI), duration of diabetes, total cholesterol (TC), fasting blood glucose (FBG), postprandial blood glucose (PBG), microalbuminuria, and left ventricular ejection fraction (LVEF); the fully adjusted model included age, sex, BMI, duration of diabetes, TC, FBG, PBG, microalbuminuria, LVEF, and left atrial conduit strain (LAScd).

### 4.4 Curve fitting

**Figure 2:**
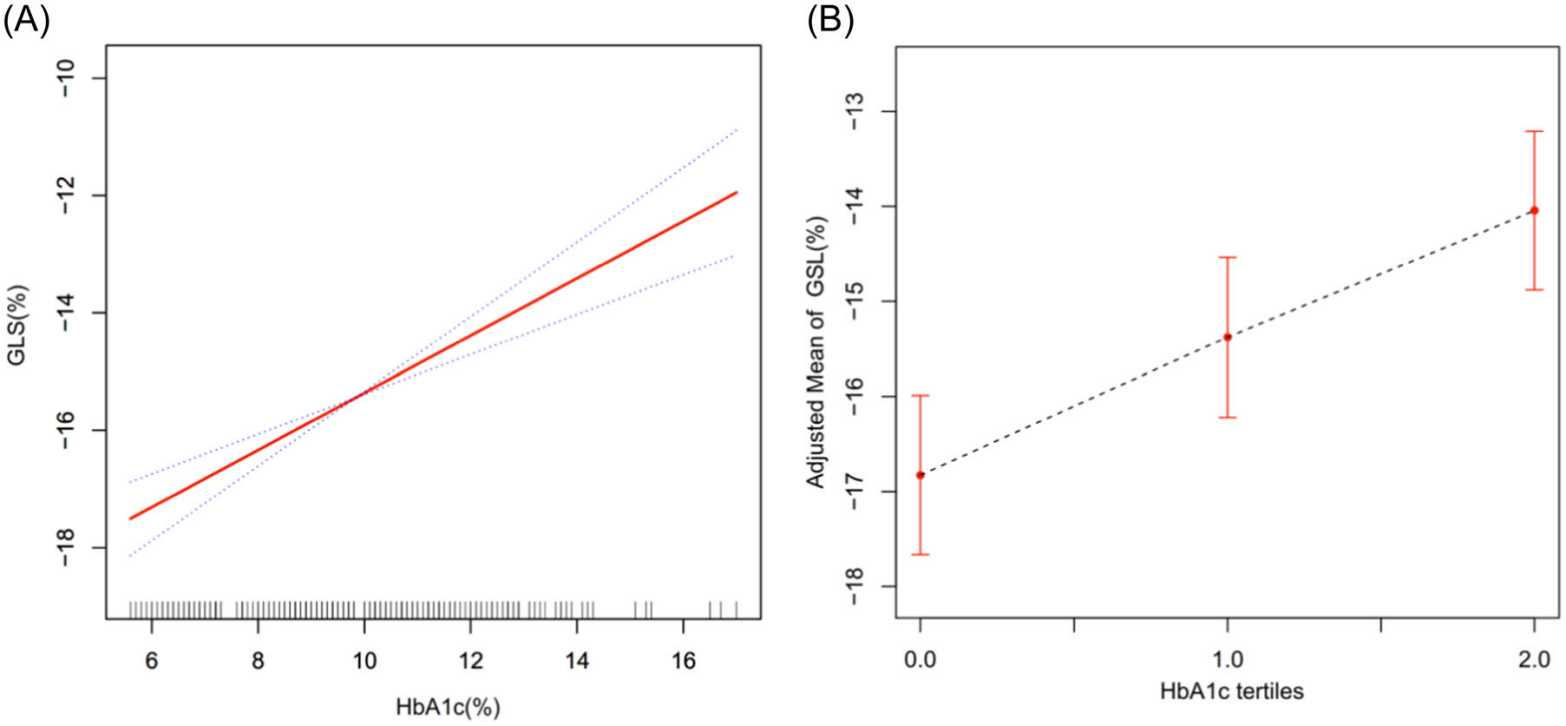
Relationship between glycated hemoglobin A1c (HbA1c) level and left ventricular global longitudinal strain (GLS). Red solid line represents the fitted line of HbA1c level and GLS, and the blue dashed line represents the corresponding 95% confidence interval.

GAM was used to examine the relationship between HbA1c level and GLS. After adjusting for age, sex, BMI, duration of diabetes, TC, FBG, PBG, microalbuminuria, LVEF, and LAScd, results of analysis revealed that, as HbA1c levels increased, GLS values also gradually increased (*df* = 1, *p* < .001, *F* = 41.533), revealing an approximately linear relationship between the two (Figure 2). GLS increased with increases in HbA1c level.

### 4.5 Intercorrelation

**Figure 3:**
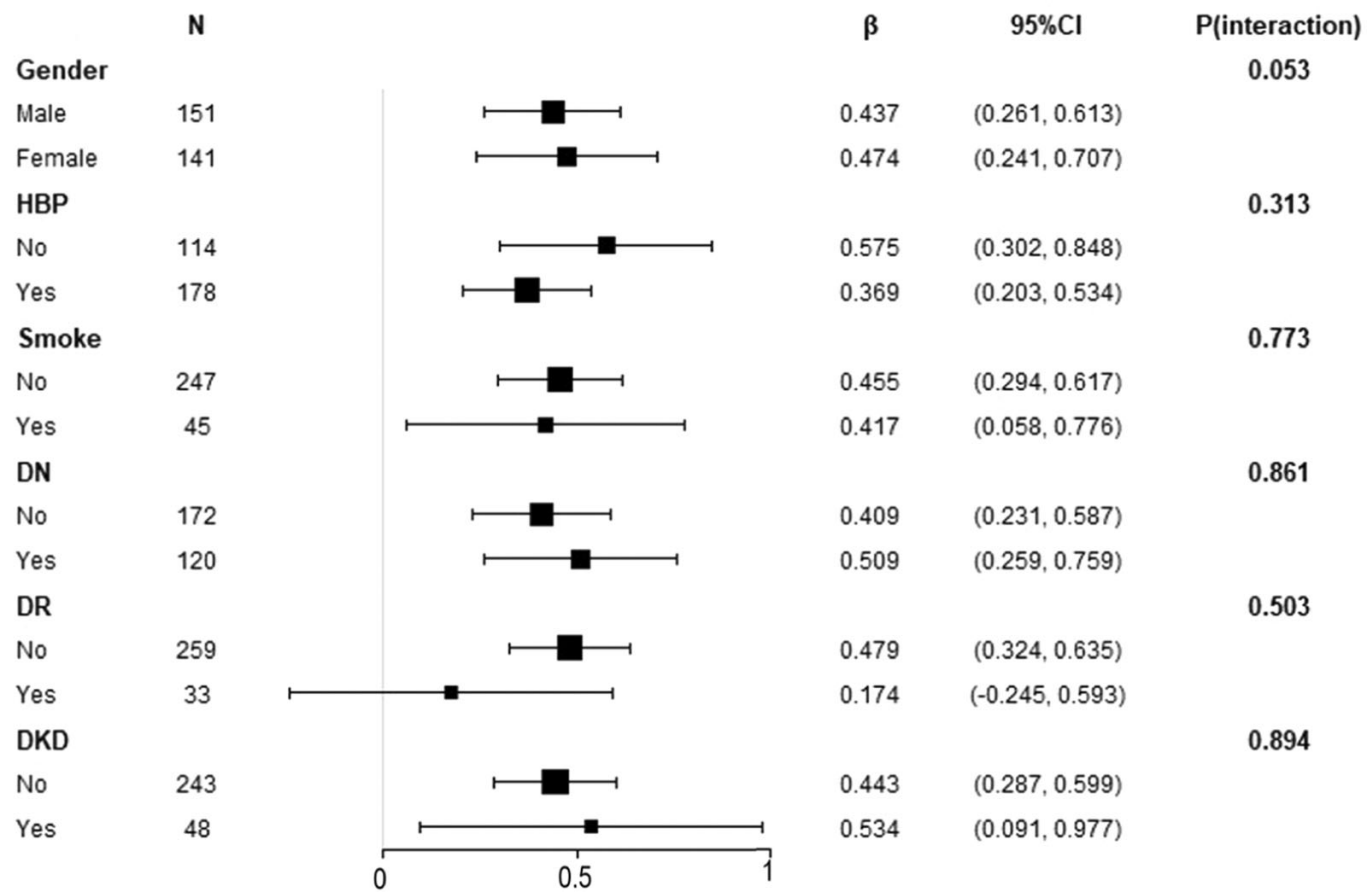
The association between global longitudinal strain and hemoglobin A1c in distinct subgroups.

The stratified analysis method was used to further assess the association between GLS and HbA1c in distinct subgroups. No variables (including sex grouping, histories of drinking, smoking, hypertension, diabetes, peripheral vascular disease, stroke or TIA, hyperlipidemia, cardiac insufficiency, and heart rate grouping) significantly altered the association between GLS and HbA1c across the various subgroups (Figure 3).

## 5 DISCUSSION

In this study, among T2DM patients with preserved LVEF, LV GLS, an essential indicator reflecting subclinical changes in the left ventricle, exhibited a positive correlation with increasing HbA1c levels and was not influenced by left atrial function. Research has demonstrated that, in most patients with diabetes and subclinical cardiac abnormalities, these abnormalities can be reversed if detected early.^3^, ^4^ Therefore, actively and effectively managing blood glucose levels, detecting cardiac abnormalities in diabetic patients early, and providing timely intervention and treatment can reduce the incidence of HF in this patient population.

T2DM is a metabolic disease caused by various factors, and is characterized by a low‐level inflammatory state and endothelial dysfunction, which increases the risk for cardiovascular disease(s). T2DM can lead to myocardial dysfunction, initially manifesting as subclinical dysfunction, and gradually developing into DCM and HF. DCM is a specific type of cardiomyopathy resulting from diffuse myocardial fibrosis, myocardial hypertrophy, and diabetic microangiopathy.^13^ There is an independent correlation between HbA1c levels and the development of HF.^14^ For every 1% increase in HbA1c, the relative risk for cardiovascular disease in patients with T2DM increases by 18%, and the risk for HF increases by 8%.^15^ Su et al.^16^ reported that, among patients with diabetes, glucose metabolism disorder(s) can manifest as significant blood glucose fluctuations, which are more harmful to the development of diabetic complications compared with continuous chronic hyperglycemia. HbA1c better reflects blood glucose levels over the previous 3 months; if blood glucose fluctuations are significant, HbA1c may increase. Studies have shown that advanced glycation end products (AGEs) and their receptors (i.e., “RAGEs”) can have adverse effects on multiple organs, including the heart,^17^ promoting the onset and progression of atherosclerosis.^14^ Additionally, hyperglycemia and poorly controlled diabetes can cause conformational changes and unfolding of some hemoglobin and increasing erythrocyte viscosity; these structural changes may lead to microangiopathy and diabetic complications.^18^ Persistent hyperglycemia will alter cardiomyocyte metabolism, damage myocardial microvessels, cause myocardial ischemia and hypoxia and, consequently, affect myocardial systolic and diastolic function. Therefore, higher levels of HbA1c are crucial indicators of cardiovascular disease and all‐cause mortality in patients with diabetes, necessitating the identification of early indicators of myocardial dysfunction in such patients.

The effects of hyperglycemia on the heart can manifest early; however, patients may not exhibit overt symptoms. Echocardiographic research involving diabetic populations primarily concentrates on LV structure and function; however, conventional parameters, such as LVEF, fail to accurately represent subtle changes in LV systolic function. Results of our study also revealed no statistical differences in conventional echocardiographic parameters, such as LVEF and E/e′ ratio, among individuals with varying HbA1c levels. Speckle tracking echocardiography is a highly sensitive technique that facilitates early detection of overall alterations in LV function. Decreased circumferential and longitudinal strain rates can be identified merely a few weeks after the onset of diabetes.^13^ Abnormal glucose metabolism‐associated subclinical left ventricle remodeling and contractile dysfunction primarily manifest as impaired longitudinal contraction and cardiac torsion.^19^ Multivariate analysis in this study also confirmed the relationship between elevated HbA1c and decreased LV longitudinal contractile function. Previous research has also suggested a correlation between GLS and HbA1c levels.^20^ Consequently, early detection of cardiovascular damage in patients with T2DM is crucial for early intervention measures and increasing awareness of the importance of blood glucose control.^21^

There is a close interaction between left atrial and LV function,^22^ with atrio‐ventricular coupling being one of the main predictors of cardiovascular morbidity and mortality in patients with diabetes.^23^ Recent studies have also underscored the clinical significance of left atrial function in this patient population.^24^ Structural, functional, and mechanical changes in the left atrium are sensitive and reliable markers of cardiac remodeling and function. Left atrial function is a vital indicator for evaluating LV filling pressure and diastolic function, and changes in atrial function precede the development of left atrial fibrosis and volume increase.^25^ Its primary function is to regulate LV filling, and also significantly impacts LV systolic function.^22^ In this study, differences in the distribution of the left atrial function index (i.e., LAScd) among various HbA1c levels were observed (*p* ≤ .05). Consequently, when studying the relationship between GLS and HbA1c level, the index LAScd may serve as an important confounding factor. After adjusting for confounding factors in this study, including the LAScd index of left atrial strain, the independent association between LV GLS and HbA1c levels was confirmed. Research by Zairi et al. demonstrated that the decline in LV GLS in children with type 1 diabetes was correlated with increased HbA1c levels.^26^ Zhou et al.^27^ discovered that elevated HbA1c levels were independently associated with decreased peak diastolic strain rate through CMR‐feature tracking. In summary, our study revealed that increases in HbA1c levels was closely associated with decreases in GLS and was not influenced by left atrial function.

There were several limitations to the present study, the first of which was the small sample size, which may not have been representative of the entire T2DM population, thus necessitating validation through larger‐scale studies. Second, the determination of LV GLS depended on the image frame rate, and image quality may have affected the accuracy of speckle tracking.

In conclusion, results of our study demonstrated that, for T2DM patients with preserved LVEF, as HbA1c levels rise, LV GLS, a crucial indicator reflecting subclinical changes in the left ventricle, was independently associated with GLS and remained unaffected by left atrial function. For T2DM patients with poor blood glucose control and substantial fluctuations, proactive blood glucose management and early intervention should be used to help delay or even reverse myocardial damage and prevent the worsening of cardiac function.

## AUTHOR CONTRIBUTIONS

Min Xu designed the study and carried out the study, data collection and analysis. Jinmei Gao wrote the manuscript and Mingxia Gong revised the manuscript. Xiaohong Jiang and Ming Chen designed part of the experiments. Shu Jiang and Zhenni Yang collected the T2DM patients. All authors read and approved the final manuscript.

## CONFLICT OF INTEREST STATEMENT

The authors declared no conflict of interest.

## Data Availability

The data that support the findings of this study are available on request from the corresponding author.
